# SARS-CoV-2 RNA in plasma samples of COVID-19 affected individuals: a cross-sectional proof-of-concept study

**DOI:** 10.1186/s12879-021-05886-2

**Published:** 2021-02-17

**Authors:** Luna Colagrossi, Maria Antonello, Silvia Renica, Marco Merli, Elisa Matarazzo, Giovanna Travi, Marta Vecchi, Jacopo Colombo, Antonio Muscatello, Giacomo Grasselli, Silvia Nerini Molteni, Vittorio Scaravilli, Emanuele Cattaneo, Diana Fanti, Chiara Vismara, Alessandra Bandera, Andrea Gori, Massimo Puoti, Valeria Cento, Claudia Alteri, Carlo Federico Perno

**Affiliations:** 1 Department of Laboratories, Unit of Diagnostic Microbiology and Immunology, Bambino Gesù Children’s Hospital, IRCCS, Rome, Italy; 2 Department of Oncology and Hemato-oncology, Università degli Studi di Milano, Milan, Italy; 3 Infectious Diseases, ASST Grande Ospedale Metropolitano Niguarda, Milan, Italy; 4 Residency in Microbiology and Virology, Università degli Studi di Milano, Milan, Italy; 5 Department of Cardiotoracovascular Anesthesia and Intensive Care, ASST Grande Ospedale Metropolitano Niguarda, Milan, Italy; 6 Centre for Multidisciplinary Research in Health Science, University of Milan, Milan, Italy; 7 Infectious Diseases Unit, IRCCS Ca’ Granda Ospedale Maggiore Policlinico Foundation, Milan, Italy; 8 Department of Anaesthesia and Critical Care, IRCCS Ca’ Granda Ospedale Maggiore Policlinico Foundation, Milan, Italy; 9 Chemical-clinical and Microbiological Analyses, ASST Grande Ospedale Metropolitano Niguarda, Milan, Italy

**Keywords:** Viremia, SARS-CoV-2, COVID-19, ddPCR, Molecular diagnosis, Hematological malignancies

## Abstract

**Background:**

Recent studies showed that plasma SARS-CoV-2 RNA seems to be associated with worse COVID-19 outcome. However, whether specific population can be at higher risk of viremia are to date unexplored.

**Methods:**

This cross-sectional proof-of-concept study included 41 SARS-CoV-2-positive adult individuals (six affected by haematological malignancies) hospitalized at two major hospital in Milan, for those demographic, clinical and laboratory data were available. SARS-CoV-2 load was quantified by ddPCR in paired plasma and respiratory samples. To assess significant differences between patients with and patients without viremia, Fisher exact test and Wilcoxon test were used for categorical and continuous variables, respectively.

**Results:**

Plasma SARS-CoV-2 RNA was found in 8 patients (19.5%), with a median (IQR) value of 694 (209–1023) copies/mL. Viremic patients were characterized by an higher mortality rate (50.0% vs 9.1%; *p* = 0.018) respect to patients without viremia. Viremic patients were more frequently affected by haematological malignancies (62.5% vs. 3.0%; *p* < 0.001), and had higher viral load in respiratory samples (9,404,000 [586,060-10,000,000] vs 1560 [312–25,160] copies/mL; *p* = 0.002).

**Conclusions:**

Even if based on a small sample population, this proof-of-concept study poses the basis for an early identification of patients at higher risk of SARS-CoV-2 viremia, and therefore likely to develop severe COVID-19, and supports the need of a quantitative viral load determination in blood and respiratory samples of haematologic patients with COVID-19 in order to predict prognosis and consequently to help their further management.

**Supplementary Information:**

The online version contains supplementary material available at 10.1186/s12879-021-05886-2.

## Background

Severe Acute Respiratory Coronavirus 2 (SARS-CoV-2) has been detected in specimens from multiple sites [1–3], including plasma. Its frequency ranges from 1 to 73% [2–11] in COVID-19 patients, and it seems to be significantly associated with adverse clinical outcomes [5, 7–10]. Preliminary data aside, the relationship between patients’ characteristics, plasma SARS-CoV-2 RNA, and the severity and outcome of COVID-19, remains largely unexplored.

In this study, we systematically quantified SARS-CoV-2 load in paired plasma and respiratory samples of 41 well characterized patients, with the aim of defining whether the presence of viremia could be correlated with clinical and/or laboratory characteristics, and COVID-19 outcome.

## Methods

### Data collection

This proof-of-concept cross-sectional study included 41 SARS-CoV-2-positive adult patients hospitalized at Grande Ospedale Metropolitano Niguarda and at Fondazione IRCCS Policlinico Ca′ Granda (Milan, Italy) since March 01, 2020, and who have either died (*N* = 7) or been discharged (*N* = 34) by July 01, 2020, for those paired respiratory (nasopharingeal swabs or bronchoalveolar lavages) and plasma samples were available. Demographic and clinical data were retrospectively collected thanks to pseudonymized electronic forms. The severity of the disease was classified in mild, moderate, severe and critical in line with WHO scale [12].

SOFA score and Charlson Comorbidity index were computed from the patients’ electronic medical records.

The study protocol was approved by the Ethical Committees of ASST Grande Ospedale Metropolitano Niguarda and IRCCS Ca′ Granda Ospedale Maggiore Policlinico Foundation (Prot. Numbers 92–15,032,020 and 0008489) and conducted in accordance with the principles of the 1964 Declaration of Helsinki. Study participation was by written informed consent. The requirement for informed consent was waived for ICU patients for those communication of information was impossible or characterized by disproportionate efforts [13].

### SARS-CoV-2 quantification

Total RNA was extracted from 280 ul of respiratory samples or plasma samples using QIAamp viral RNA mini kit (Qiagen) following manufacturer’s instruction. SARS-CoV-2 genomic RNA was quantified by means of the QX200™ Droplet Digital™ PCR System (ddPCR, Biorad) using an home-made protocol targeting the RNA dependent RNA polymerase (RdRp) of SARS-CoV-2 and the housekeeping gene RNAse P [14, 15]. All results were further confirmed by using a second assay adapted for ddPCR and targeting two different portions of RdRp (https://www.who.int/docs/default-source/coronaviruse/real-time-rt-pcr-assays-for-the-detection-of-sars-cov-2-institut-pasteur-paris.pdf?sfvrsn=3662fcb6_2). SARS-CoV-2 quantification was finally expressed in number copies/mL.

### Statistical analysis

Descriptive statistics are expressed as median values and interquartile range (IQR) for continuous data and number (percentage) for categorical data. To assess significant differences between patients with and patients without viremia, Fisher exact test and Wilcoxon test were used for categorical and continuous variables, respectively.

SPSS statistical software (version 23.0, IBM, Armonk, NY, USA) was used for statistical analysis.

## Results

### Patients’ characteristics

From March 01 through July 01, 2020, a total of 1801 patients received a diagnosis of SARS-CoV-2 infection at two of major hospital in Milan, Lombardy. Forty-one patients with available paired respiratory and plasma samples for SARS-CoV-2 detection were included in the analysis. Six of them (14.6%) were affected by haematological malignancies. Demographic, clinical, and laboratory, findings at admission, stratified by presence of SARS-CoV-2 in plasma, are reported in Table 1.

**Table 1:** Demographic, clinical, viral, and laboratory findings of 41 COVID-19 patients for those blood and respiratory specimens were available for SARS-CoV-2 detection

	Overall, ***N*** = 41	Sars-CoV-2 in the blood		***P-value***
		Present, N = 8	Absent, ***N*** = 33	
**Demographics and clinical characteristics**				
Age, years	59 (50–67)	65 (41–69)	59 (50–66)	0.469
Sex				
*Male*	13 (30.2)	5 (62.5)	23 (69.7)	0.692
Symptoms				
*Fever*	35 (85.4)	5 (62.5)	30 (90.9)	0.077
*Cough*	24 (58.5)	3 (37.5)	21 (63.6)	0.172
*Dyspnea*	28 (68.3)	5 (62.5)	23 (69.7)	0.692
Evidence of interstitial pneumonia	39 (95.1)	7 (87.5)	32 (97.0)	0.356
Chronic comorbidities	27 (65.9)	6 (75.0)	21 (63.6)	0.435
*Solid malignancy*	5 (12.2)	1 (12.5)	4 (12.1)	0.683
*Haematological malignancies*^a^	6 (14.6)	5 (62.5)	1 (3.0)	**< 0.001**
*Diabetes*	5 (12.2)	2 (25.0)	3 (9.1)	0.246
*Obesity*	9 (22.0)	0 (0.0)	9 (23.7)	0.116
*Cardiovascular disease*	3 (7.3)	2 (25.0)	1 (3.0)	0.092
*Chronic obstructive lung disease*	4 (9.8)	1 (12.5)	3 (9.1)	0.596
*Hypertension*	8 (19.5)	1 (12.5)	7 (21.2)	0.503
*Chronic kidney disease*	1 (2.4)	0 (0.0)	3 (9.1)	0.512
*Chronic liver disease*	1 (2.4)	0 (0.0)	1 (3.0)	0.805
*Other*^b^	3 (7.3)	0 (0.0)	3 (9.1)	0.512
Immunosuppressive therapy	27 (65.8)	4 (50.0)	23 (69.9)	0.411
SOFA score^c^	3 (1–4)	2 (1–4)	3 (1–4)	
0	5 (12.8)	0 (0.0)	5 (15.6)	0.828
1–2	12 (30.8)	4 (57.1)	8 (25.0)	
3	8 (20.5)	1 (14.3)	7 (21.9)	
≥ 4	14 (35.9)	2 (28.6)	12 (37.5)	
CHARLSON comorbidity index	2 (1–4)	4 (2–6)	2 (1–3)	
0	7 (17.1)	1 (12.5)	6 (18.2)	0.298
1–2	19 (46.3)	2 (25.0)	17 (51.5)	
3–4	8 (19.5)	2 (25.0)	6 (18.2)	
≥ 5	7 (17.1)	3 (37.5)	4 (12.1)	
COVID-19 manifestation				
*Critical*	15 (36.6)	4 (50.0)	11 (33.3)	1.000
*Severe*	14 (34.1)	2 (25.0)	12 (36.4)	0.692
*Moderate*	11 (26.8)	2 (25.0)	9 (27.3)	1.000
*Mild*	1 (2.4)	0 (0)	1 (3.0)	1.000
Time from symptoms-onset to hospital admission, days	5 (3–7)	3 (1–5)	6 (3–8)	**0.024**
**SARS-CoV-2 load, ddPCR**				
Respiratory samples^d^				
*Viral load, copies/mL*	3680 (595–166,800)	9,404,000 (586,060-10,000,000)	1560 (312–25,160)	**0.002**
Blood samples				
*Viral load, copies/mL*	0 (0–0)	694 (209–1023)	0 (0–0)	–
**Laboratory findings**^e^				
White blood cell count, × 10^9^ per L	8.43 (5.59–11.58)	9 (2.70–9.90)	8.30 (5.70–12.50)	0.295
Lymphocytes count, × 10^9^ per L	0.74 (0.55–1.06)	0.50 (0.40–1.00)	0.80 (0.60–1.10)	0.230
Platelet count, × 10^9^ per L	155 (78–274)	108 (77–285)	202 (78–274)	0.567
Total bilirubin, mg/mL	0.47 (0.28–0.66)	0.40 (0.20–0.80)	0.50 (0.30–0.60)	0.574
Estimated glomerular filtration rate, mL/min	83 (57–99)	57 (46–105)	88 (62–98)	0.806
Creatinine, μmol/L	85 (70–107)	107 (76–132)	82 (69–98)	0.264
D-dimer, μg/mL	1.08 (0.70–2.11)	1.20 (0.80–3.20)	1.10 (0.70–1.90)	0.629
IL-6, pg/mL	53 (16–203)	62 (28–134)	44 (15–251)	0.968
Lactate dehydrogenase, U/L	320 (220–422)	236 (220–332)	344 (217–552)	0.252
**Outcome**				
Death	7 (17.1)	4 (50.0)	3 (9.1)	**0.018**
*Time from symptoms-onset to death, days*	36 (27–83)	31 (21–42)	83 (28–98)	0.400
Discharged	34 (82.9)	4 (50.0)	30 (90.9)	**0.018**
*Time from symptoms-onset to discharge, days*	50 (33–73)	38 (35–39)	54 (30–77)	0.621

Data are expressed as median (interquartile range), or N (%). *P*-values comparing COVID-19 patients with evidence of RNAemia to patients without RNAemia were calculated by Mann-Whitney U test, or Fisher’s exact test, as appropriate. *P* < 0.05 was considered statistical significant. ALT = alanine aminotransferase. AST = aspartate transaminase. COVID-19, coronavirus disease 2019. ^a^Including: Large cell lymphoma (n = 4), T-cell lymphoma (n = 1), Hodgkin’s Lymphoma (n = 1). ^b^Including: Sarcoidosis (*n* = 1), Hashimoto’s thyroiditis (n = 1), renal transplantation (n = 1). ^c^Information available for 39 individuals. ^d^ 20 Bronchoalveolar lavages and 21 nasopharingeal swabs. SARS-CoV-2 load did not differ between the two different samples (median, IQR: 3680, 366–166,800 vs 1530, 249–11,230, *P* = 0.419). ^e^Information available for 40 individuals

Thirteen patients (30.2%) were male, and median age was 59 (IQR: 50–67) years. Chest radiographs or CT scan confirmed a classical bilateral interstitial pneumonia in 95.1% of cases (39/41). Looking at the outcome available, 7 patients (17.1%) died in a median time of 28 (27–36) days from symptoms onset, and 34 (82.9%) were discharged in a median time of 54 (30–76) days from symptoms onset at the study endpoint.

### Sars-CoV-2 load in respiratory and plasma samples

The presence of SARS-CoV-2 RNA in respiratory specimens was confirmed for all 41 patients, with a median (IQR) viral load of 3680 (595–166,800) copies/mL, with no difference between the bronchoalveolar lavages and nasopharingeal swabs (median, IQR: 3180 [312–166,800] vs 9040 [671–6,800,000] copies/mL, *p* = 0.149). SARS-CoV-2 load was found in 8 plasma samples (19.5%), with a median (IQR) value of 694 (209–1023) copies/mL (Table 1).

Stratifying the population by plasma SARS-CoV-2 RNA, viremic patients were characterized by an higher mortality rate (50.0% vs 9.1%; *p* = 0.018) respect to no viremic patients. Respiratory failure was the leading cause of death in 3 out of 4 patients with SARS-CoV-2 viremia and in the 2 out 3 patients without evidence of SARS-CoV-2 in plasma. The underlying disease found in the remaining death with viremia was a T-cell lymphoma at advanced stage.

By looking at the characteristics of patients with viremia, we found that they were more frequently affected by haematological malignancies (5 [62.5%] vs. 1 [3.0%]; *p* < 0.001), and had higher viral load in respiratory samples (9,404,000 [586,060-10,000,000] vs 1560 [312–25,160] copies/mL; *p* = 0.002).

### SARS-CoV-2 dynamics in the 8 patients with SARS-CoV-2 viremia

**Fig. 1:**
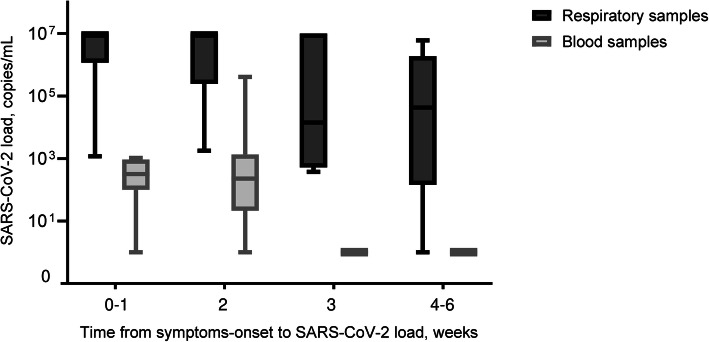
Decay of SARS-CoV-2 load in respiratory and blood samples of the 8 viremic patients, against time from symptoms onset. Box and whiskers plots indicates the distribution of SARS-CoV-2 load according to sample (respiratory vs. blood) and time from symptoms-onset. The upper and lower quartiles are represented by boxes, while the variability outside this range by whiskers. The median value is represented by a short line within the box

SARS-CoV-2 loads in plasma and respiratory samples of the 8 viremic patients in relation to day of symptoms onset was monitored until viremia clearance (defined as two consecutive negative plasma samples, Fig. 1, number of samples per patient median, [IQR]: 4 [2–6]). Viremia was detected since 12 (9–14) days after symptom onset, and viral clearance occurred while SARS-CoV-2 in respiratory samples was still detectable (SARS-CoV-2 load copies/mL median, [IQR]: 1,607,260 [1418-8,303,100]). Two patients died while still viremic.

### Focus on hematological malignancies

As mentioned before, 5 out 6 patients affected by hematological malignancies were viremic for SARS-CoV-2 (Supplementary Table 1). Three of these 5 viremic patients were affected by large B-cell lymphoma (60.0%) and 4 received chemotherapy during COVID-19 (80.0%), with one patient receiving Bendamustine in combination with Gemcitabine and Vinorelbine (BEGEV). The median time to SARS-CoV-2 clearance in respiratory tract and plasma was of 6.5 (4.4–6.5) and 1.7 (1.5–3.0) weeks, respectively. The longer time for viral clearance in respiratory and peripheral tracts was observed in one patient affected by Hodgkin lymphoma and treated with BEGEV, followed by one patient affected by diffuse large B-cell lymphoma with a recent treatment history of high-dose methotrexate.

## Discussion

In our study plasma SARS-CoV-2 RNA was found in 8 (19.5%) individuals, 6 (75.0%) of whom had a severe or critical COVID-19 manifestation and 4 (50.0%) died. This is in line with studies published so far, describing plasma SARS-CoV-2 RNA detectable in different percentage of COVID-19 patients, ranging from 0 to 73%, and frequently and significantly associated with worse clinical outcomes [2–11]. It should be noted that SARS-CoV-2 was not the first respiratory viral agent to be detected in blood of critical individuals. The viremic phase was frequently correlated with the severity of the disease and worse outcome in most viral infections, like those sustained by Rhinoviruses, Respiratory syncytial virus, Adenovirus, SARS-CoV, and Metapneumovirus [16–18].

Now that more evidence suggests a close relationship between viremia and severe COVID-19, the identification of specific factors or conditions that can define a population at risk of viremia is of outmost importance. In our study, plasma SARS-CoV-2 load was frequently detected in patients affected by hematological malignancies (62.5% vs. 3.0%, *p* < 0.001), and with higher viral load in respiratory samples (9,404,000 [586,060-10,000,000] vs. 1560 [312–25,160] copies/mL, *p* = 0.002), as compared to patients without viremia (Table 1). Previous studies on series of hospitalised patients showed that the concurrent haematological malignancies and COVID-19 are strongly related to mortality [19], and to prolonged viral shedding in the respiratory tract and serum samples [1, 20]. An episode of prolonged plasma SARS-CoV-2 RNA started prior to the onset of symptoms and diagnosis of COVID-19 was also recently described in a 51-year-old patient with acute myeloid leukemia, and thus with a heavily compromised immune status [21]. Consistent with these earlier reports, we found that viremia was detected since 12 (9–14) days from symptom onset, and viral undetectability in the blood occurred while SARS-CoV-2 in respiratory samples was still present. All these evidences may pose this fragile population at higher risk of developing a systemic infection, suggesting a potential utility of plasma SARS-CoV-2 RNA testing as a prognostic indicator in severe immunocompromised patients.

Our proof-of-concept study has some limitations. a) The small sample size and the high proportion of individuals with chronic comorbidities (80.5% of patients) prevented to appreciate a clear association between viremia and severe COVID-19, that preliminary data have already shown [7–10], as well as correlations between viremia and IL-6 or other knows COVID-19 prognostic markers (i.e. lymphocytes count, lactate dehydrogenase or D-dimer level) [5, 22, 23]. b) The correlation between viremia and the outcome was based only on 4 definitive events, requiring a confirmation on larger sample size. c) The real infectivity of the virus found in plasma was not assessed. Thus, it cannot be determined at present whether detectable RNA represents intact infectious virus or inactive, non-replicating nucleic acid. d) The study design, the heterogeneity of hematological malignancies and chemotherapies prevented to draw certain conclusions about correlations between these parameters and time to SARS-CoV-2 clearance. Even if the prolonged respiratory shedding and persistent viremia observed in one patient treated with the bendamustine is in line with recent findings, [24, 25] this observation requires further evaluation by ad hoc designed studies.

## Conclusion

Our cross-sectional proof-of concept study provides evidence for plasma SARS-CoV-2 load detection in the 19.5% of patients (*n* = 8), 50% of whom died, confirming the association between viremia and worse outcome [7–11]. Patients with SARS-CoV-2 in plasma were frequently characterized by haematological malignancies, and elevated viral load in respiratory samples. Although the associations here observed are based on a small population and need to be confirmed on a larger sample size, they pose the basis for an early identification of patients at higher risk of SARS-CoV-2 viremia, and therefore likely to develop severe COVID-19. This proof-of-concept study also supports the need of a quantitative viral load determination in blood and respiratory samples of haematologic patients with COVID-19 in order to predict prognosis and consequently to help their further management.

## Supplementary Information

**Additional file 1.**

## Data Availability

Data are described in Table 1, Supplementary Table 1 and Fig. 1. The datasets used in this study will be available from the corresponding author on reasonable request.

## Abbreviations

SARS-CoV-2: Severe Acute Respiratory Coronavirus 2
COVID-19: COronaVIrus Disease 2019
ARDS: Acute Respiratory Distress Syndrome
ddPCR: Droplet Digital PCR
RdRp: RNA dependent RNA polymerase
