# Supplementary material for: SARS-CoV-2 RNA in plasma samples of COVID-19 affected individuals: a cross-sectional proof-of-concept study
Source: BMC Infect Dis. 2021 Feb 17;21:184. doi: 10.1186/s12879-021-05886-2 (PMC7887543; doi:10.1186/s12879-021-05886-2)
Supplement: Supplementary file 1 — Additional file 1. [file 12879_2021_5886_MOESM1_ESM.docx]

**Supplementary Table 1.** Clinical characteristics of the six patients affected by hematological malignancies.

| **ID** | **Hematological Malignancy** | **Chemotherapy during COVID-19** | **Baseline SARS-CoV-2 RNA in respiratory samples^a^, copies/mL** | **Baseline SARS-CoV-2 RNA in plasma samples, copies/mL** | **Time to SARS-CoV-2 clearance in respiratory tract^b^, weeks** | **Time to SARS-CoV-2 clearance in plasma, weeks** |
| --- | --- | --- | --- | --- | --- | --- |
| 1 | Diffuse large B-cell lymphoma | R-DHAP | 5,720 | 924 | 4.4 | 1.0 |
| 2 | Hodgkin lymphoma | BEGEV | >10^7^ | 1,044 | 8.6 | 3.0 |
| 3 | Diffuse large B-cell lymphoma | none | >10^7^ | 0 | 6.5 | - |
| 4 | Diffuse large B-cell lymphoma | R-COMP | >10^7^ | 1,016 | na* | 1.5 |
| 5 | Peripheral T-cells lymphoma | CHO(E)P | 1,166,400 | 80 | 3.8 | 1.7 |
| 6 | Diffuse large B-cell lymphoma | none^ | >10^7^ | 464 | 6.5 | 3.4 |

R-DHAP: rituximab, dexamethasone, cytarabine, ciSplatin; BEGEV: bendamustine, gemcitabine, vinorelbine; R-COMP: Nonpeghylated liposomal doxorubicin combination regimen; CHOEP: cyclophosphamide, doxorubicin, etoposide, vincristine and prednisone.

^a^ All nasopharyngeal swabs.

^b^ Defined by persistence of SARS-CoV-2 in nasopharyngeal swabs.

^ High-Dose (HD) Methotrexate within 4 months before COVID-19 diagnosis.

*Patient died while still viremic.
